# Enhancing the digital adoption in healthcare built environments: a framework for a therapeutic user-based wayfinding system

**DOI:** 10.3389/fmedt.2025.1323446

**Published:** 2026-02-03

**Authors:** Yasmin Garcia-Sterling, Karim Farghaly, Michael Pitt

**Affiliations:** Bartlett School of Sustainable Construction, University College London, London, United Kingdom

**Keywords:** wayfinding, user-based, healthcare, built environment, wellbeing

## Abstract

The efficacy of wayfinding systems in healthcare settings holds significant implications for many users, including patients, visitors, and healthcare professionals. Traditional systems, anchor examples in spatial design principles, frequently overlook the variegated needs of individuals, especially those with physical and mental disabilities. This narrow focus culminates in a one-size-fits-all approach that fails to address the intricate diversity of user requirements, thereby compromising sensory spatial information that aids a user's real-time neurophysiological needs. To rectify this, the narrative review paper reviews the potential contribution of the ecology of wayfinding for understanding healthier wayfinding solutions. It proposes a paradigm shift towards a user-centric wayfinding model in healthcare facilities. Building on a nuanced understanding of the diverse needs and neurological factors affecting healthcare users, this paper describes and synthesises elements that adapt to the triangulation from the Human Activity Accessible Technology, (HAAT) to understand hospital users' wayfinding needs at a temporal to spatial level. Lastly, anticipating the novel conceptual framework to be considered a curb-cut effect for a therapeutic, user-based wayfinding system, beyond the assistive technology phenomenon. Adopting a humanistic lens, the framework aims to elevate the well-being and comfort of users by incorporating inclusion criteria into digital wayfinding systems. The paper concludes by identifying areas for future research to integrate user-based real-time capabilities into healthcare wayfinding technology, thereby aligning therapeutic objectives with an inclusive and supportive healthcare environment.

## 1 Introduction

Wayfinding can be defined as the experience a user encounters through a journey to reach a destination (1). UK standards such as PAS 6463-2022 (2), Design for the Mind (3) and Dementia Friend Design Tool (4) highlight that when a destination is not easily identifiable, real anxiety can be foreseen as an unsettling part of the experience (5). Wayfinding is the medium to respond to user needs through complex analysis and is normally considered successful when it is invisible in its physical operation (6). Otherwise, wayfinding can induce stress, high blood pressure, physical aggression, and fatigue (7–9, 146). Particularly in complex buildings such as hospitals, and especially when the user is ill and has added time constraints, polarising impaired information processing can be triggered by stress (6, 10). Under environmental constraints, we must question how a healthful wayfinding process can remain efficiently possible. Compared to contemporary spatial designs as predicted, Schneider (11) highlights those sensory modalities that shape perception and play an important role in how users cognitively interpret the surrounding environment. In contribution, exploring the relationship between users' sensory information awareness and the potential for inducing well-being in the design experience is further reviewed.

One of the benefits of wayfinding systems using broader service methods is the involvement of user diversification. The embodying of heterogeneous properties from the end-users, contribute to the responsibilities of the therapeutic built environment, which may be included across the building life cycle (6, 12). These subjective results align with substantial healthcare time-saving contributions (13), as an inefficient cost spent in hospitals from late appointments and no-shows that have been affecting the healthcare bottom line, since the 1990s (14–16). Ultimately, with the purpose to bring back this body of wayfinding experience, alongside todays digitally applied interventions are to be considered as a first step in developing diversification as a research reference for sourcing a therapeutic wayfinding system.

It is without withstanding that innovations in personalisation for spatial design and other sensory information arrangements are receiving growing attention in academic discourse (17). However, these advancements are rarely applied in the context of wayfinding. Only a few studies address these thematics through this lens (11, 18), and these are beyond specific assistive technologies that support navigating in a practical context. Due to the growing complexity of healthcare infrastructure, there is a call for more research in wayfinding management to aid in creating a therapeutic environment. Such understanding is crucial for supporting accessibility across a diverse range of user needs, from physiological (such as anxiety, space confusion, and disorientation) to cognitive (like processing, bias, and coping) and behavioural factors (including information availability, environmental hazards, and physical mobility), considering both spatial and temporal constraints.

This study suggests that the evolving information requirements of users and the types of dynamic information that can better address these user concerns in real time should be recognised. Signified by the various spatial constraints in healthcare delivery to propose an agency-dominated system for wayfinding management, moving beyond traditional spatial planning methods of mapping design today. In this initial section of the paper, we commence by presenting an overview of the interconnection between the user and the spatial environment, followed by a triangulation of theories can that guide our investigation. Presenting the eight categories that comprise our model discussing them under healthcare wayfinding activities apparent across social and temporal dimensions. Lastly, we conclude this section with a roadmap for future research, delineating how each task aligns with pertinent supporting materials, thus paving the way for the adoption of technological advancements in wayfinding within the realm of a therapeutic healthcare-built environment.

## 2 Wayfinding phenomenology in healthcare

Wayfinding first evolved through Kevin Lynch's seminal (19), 1960 study on spatial navigation and although has a historical lineage that traces back 500 years. Wayfinding is referred to the techniques used for locating unknown or mislabelled routes and ongoing maintenance. It was formerly defined as “a consistent use and organisation of definite sensory cues from the environment” (*ibid*). In the revolution for signage followed in the 1970s, the Society for Environmental Graphic Design (SEGD) designers shifted from an artistic architectural vision towards integrating function into physical space for user orientation (20). Since then, wayfinding became referred to as “information systems”, and has advanced its definition to “strategically connect individuals to a physical place, constituted by a sign system and designed as a network” (21). Both Weisman (22) and, later, Garvey (23) has verified wayfinding as a principle for legible recognition to connote experiences between destinations.

Although, studies by ecologists indicate that the relationship between an individual and a space is fundamentally tied to the innate fear of becoming disoriented or lost (24). Fundamental to human history, venturing builds on our survival system and the need to find necessities such as food, water, companionship, and returning home, all specific to survival (*ibid*). Historically, and before the term “wayfinding” was coined, finding a way through space consisted of instincts apparent to natural orientation and involved developing a spatial understanding of natural patterns, instincts, and phenomena. These include watching bird behaviour, cloud pattern movement, wind direction, and sunlight (25, 26). Creating a route from the surroundings reveals “signatures of the motion” (27), and as Gooley (27) continues to observe, having an awareness of the outdoors becomes primary to conscious thought. However, during the Industrial Revolution, the built environment was “*designed*” and so were the series of cues users required to move from one place to another (28). Consequently, wayfinding faced influence in acquired information and shifted from the process of executing complex mental operations to deduction and logic. Preziosi (28) contends that signage became homogenous “since each belongs to and is defined principally in relation to, the overall system of signs of which each is an exemplar or material realisation”. This shift necessitated by the increase in complicated buildings, places, and spaces impacted how users are required to navigate.

As the built environment grew, an increasing presence of problems emerged for users to acquire efficiency in wayfinding methods, strategies, and systems. The increased square footage for highly used spaces such as hospitals implies greater complexity as well as a requirement for cues to inform visitors of the new surroundings and to familiarise themselves (29). Following its first “signs” healthcare publication, “HTM 65 Signs” as a recommended navigational guidance to enhance accessibility to the end-user destinations (30); important landmarks for orientation (31); HTM 65 “Signs” superseded to Wayfinding in 2005 where a legible hospital layout is recognised for human interaction, comfort, safety, and accessibility (32).

Although, over time, hospital spaces have grown increasingly complex and while guidelines been updated accordingly (33, 45) a review should be in order. Generally, advantages in complex building wayfinding information comes from situational modelling, known as a systematic signage composition, that ensures a person becomes familiar with the target information (34). This includes concepts such as “cognitive commonness” (35, 36) to describe the value in picture names to identify how signs could be designed to become more easily interpreted (37). However, considering the philosophy of universal design for the “widest range of users in mind” (38), against the focus on achieving efficiency in wayfinding awareness, the varied user health needs and the importance of creating distinctiveness (12), continues to be a subject of debate.

### 2.1 Exploring contemporary wayfinding efficiency and enhancing the user experience

Healthcare wayfinding in academia revolves around systems that indeed require efficiency (39, 40), specifically hospital accessibility (41). For example, Pouyan (40) identifies sign arrangement (visual access to signage) and a growing attention emerging to consider the legibility of the building (spatial circulation, landmarks, and layout) as crucial. As accessibility and find-abilities increase a user's functionality within a space, the end user's working memory requires a sufficient combination of capacity, speed, knowledge, and available strategies critical to learning (42). Ultimately, the indication of efficiency lies in the swiftness and precision of the person to select corridors that lead to the desired destination (43). As hospital wayfinding highlights the time sensitivity of situations, it could be argued that such pressures have decreased the users' perception of their ability to find their way. Cornell et al. (43) studies also show that self-rating has a positive correlation to the individual's overall performance. At this point, we must therefore question: can hospital wayfinding rely on users to perform a fast-thinking process universally under these environmental pressures? Similar to the Nudge theory (44), decision-making is instinctive and urges that a space design with choices should be based on the user's self over how “authorities” believe people to think – constituting hospitals to rethink agency in wayfinding efficiency needs.

With a diverse range of healthcare users, it is necessary to have a system that reduces stress, thereby creating a more favourable environment with less anxiety and, in turn, enhancing the overall efficiency of the wayfinding process. Semiotics, in this context, helps us understand signs, symbols, and other cues to convey different meanings. As a basic principle, the creation of maps used within hospitals has been utilised as the primary source for internal representations, found within appointment letters, on-site directories, and the standard operating procedure applied from staff-to-patient communication (45). Maps, generally, are used to mirror the same principles found for forming cognitive maps (46–49), thereby creating recognisable models of the world and facilitating understanding of spatial relations. The ease with which healthcare maps convey structure, fluidity surrounding points of interest, and idealistic user routes depends on the spinal points of the various zones in a building. However, all depends on the user's mental rotation to facilitate map reading and maze learning (50, 51), which is also dependent on the design of the map to sufficiently integrate and interconnect memories (52, 53). Critical to the networks within the Reference Frames theory (54) as the underlying representation explaining the translation of maps, interconnected reference frames must be made by the interpreter (*ibid*). Within large-scale spaces, the user's memory appears to play a significant role in integrating spatial and non-spatial information, highlighting it as a primary responsibility in the wayfinding process. However, a lack of clear healthcare case study evidence exists today regarding the physiological and psychological signals of wayfinding. Until then the inferring environmental awareness and understanding of the users' spatial behaviour in comprehending and interpreting effective directions remains vague.

Similarly, environmental learning is a black box in wayfinding relying on deciphering egocentric references with allocentric information to anchor the structure of one's cognitive map (55). Mou and McNamara (56) highlight that humans tend to rely more on cognitively processed and recalled information as the environment's intrinsic frame of reference rather than on supplemental aids. Since our experiences in familiar or partially familiar environments play a more significant role in navigation than relying solely on supplemental data like maps. Crucially, when building knowledge in spatial representation, basic senses and sensory-motor or proprioceptive experiences are vital to identify, encode, and store environmental knowledge. Sensory inputs, such as vision, hearing, touch, and proprioception (awareness of body position and movement), provide the foundation for understanding the spatial relationships between objects, landmarks, and oneself in the environment (57). Should a user have a limited understanding of objects, their ability to navigate within a local area may become disoriented, even if they possess a general understanding of the overall map. Ultimately, having the ability to explore an area through sensory input, speed rotation, and orientation demonstrates wayfinding as a “purposive, directed, and motivated activity” that enables individuals to navigate and understand their surroundings (58).

Alas, increased efficiency and accuracy should be coupled with fostering a deeper sense of connection and engagement with the environment. Extending a criterion for encompassing safety and legibility to serve as variables that determine a route learnt through guided movement and organised decision-making forming cognitive patterns. For example, Rodriguez et al. (59) found the presence of sidewalks to destinations, created options in route choices and proved paramount to users sourcing healthier decisions and perceived user safety. Similarly, the access to cartographic and map reading requires a logic to understand and estimate distance as an appreciation for ways space can support users. Particularly the feeling of enhanced engagement with safety in conjunction with engaging the brain to represent a spatiotemporal element that patches together experiences for a memory of learnt paths (60). Both, facilitating wayfinding's ability for exploration, links with providing emotional support, boosting confidence and offering practical assistance aiding in the recovery or rehabilitation process.

Where wayfinding then nurtures their navigation skills and overall well-being (61), sourcing research tailored to a healthcare setting has been condemned as time-consuming (62). Despite this, the focus for future research should be to explore user behaviour cues, specifically what behavioural information is required and reviewed for hospital wayfinding learning (62, 63). Particularly if conditions of visibility in wayfinding guidance are fixed, learning about patients' visual wayfinding experience (without the various stimuli that are later searched, selected, and used) remains unknown (62, 64). Ultimately, exploring wayfinding to aid the spatial environment helps users determine where users are and where they want to go. As the link with maps shows that users develop growth in neural pathways for the concept of mental maps to increase, physiological complexity with system adaptability remains stagnant. Behavioural data followed by differences in stress levels, mental fatigue levels or confusion levels (65) and an innate sense of direction, combined with active engagement in the space tested under real-world conditions (*ibid*), could prove valuable. Thereby searching for user experiences in exploring the spatial environment is the second half of this literature. By fostering the need for a direct impact on the user's internal movement abilities for navigating and finding a way through unfamiliar areas, the technical shift considers the opportunities to source computational capabilities and greater intervention opportunities.

### 2.2 Technology shifting user-based compositions in contemporary wayfinding

In the last 15 years, technologies have shifted wayfinding, resulting in a heightened socio-technological push (66). Defined as a channel that maximises the freedom a user has, capturing information from analytics to automate personalised route navigation and maintenance (67). The impacted connectivity has solidified the gap between “the environment and the observer”, adding greater value to the asset and lessening any likelihood of disorientation (68, 69). However, Sielker (70) states that data has been used significantly less for planning and design than in other fields. With 25% of software becoming location-aware (71), areas that have largely benefited include retailers using app technology to text users when they are near businesses, a practice known as geofencing. Additionally, social networking involves purging calls and mapping locations using digital information, a technique known as geolocation (72). Ultimately, geospatial data is used by users to seek and integrate their experiences, as location-based services from wayfinding technology solutions thrive, then navigation information with personalised locators, and instant communication synergise (73).

Rather than a universal system, tailoring experiences (74) is a preconditioned and smart choice to support the digitalisation of complex buildings (75). Information can personalise experiences through user data inputs, such as habits and movements (76). Nonetheless, digital wayfinding is largely impacted by outdoor route navigation systems which offer directions based on cartographic information leveraged from increasing data, consequently disrupting economies (*ibid*). In contrast, an interactive relevance within indoor public areas has yet to be unveiled (77). One contributing factor is that SEGD (20) identifies digital solutions as being primarily commercial, promotional, and advertising-led; consequently, the initial investment remains high. Decisively, their fee-dependent system hinders the digitalisation of “information commons” in other settings (70). Therefore, converting indoor universal systems to optimise user experiences necessitates considering their economic investment value. Yet, considering the recent global pandemic, geospatial power is drawing attention, the UK's Geospatial Commission, established in 2019, has negotiated a £1 billion investment over the next 10 years to promote the delivery of public services. Both effective and efficient to fostering innovation and facilitating economic growth. Including, the international advisory board for the UN World Geospatial Information Congress has made a call for international research (78, 148) and aim to diversify internationally by 2022 (79).

In reflection, the world was considered a static physical location within a community; now, it is tied to our current geolocation wherever we happen to be. To capitalise on this, the geospatial strategy of the UK for the period from 2020 to 2025 emphasises the significance of harnessing the potential of location data in the healthcare sector. The strategy aims to leverage geolocation technologies to enhance decision-making, optimise resource allocation, and foster innovation in healthcare delivery (78). It highlights the transformative impact of geospatial information in improving healthcare services, planning, and response to public health crises (*ibid*). As wayfinding will be greatly enriched by the intricacies of user-space visibility, specifically by recognising a user's propensity for making mistakes. Using geolocation to correct blind spots in movement complements the recent literature and commends the need for healthcare wayfinding as a proactive and pre-planned activity (80). Evolving the nature of user positioning and navigation - from a one-to-many approach to a personalised one-to-one experience - indicates a shift towards individualised interactions and tailored journeys for users. Examining where and inputting coordinates through a myriad of touchpoints in multiple channels, thus allows for dynamic navigation that adapts to the specific needs per user.

Replacing traditional static cues, a personalised approach offers several advantages. For example, hospitals can achieve improved patient satisfaction and operational efficiency by personalising experiences. Helping patients and visitors find their way easily, thereby reduces stress and improves the overall healthcare environments satisfaction (81). Moreover, hospitals can leverage real-time capabilities to optimise workflows, such as guiding staff members to specific locations or notifying them about critical events or emergencies. Realsing the vision for a learning health system that can contribute to minimising disruption such as, staff burnout, financial outlook, and equity suggests that digital health should be used to combat such challenges (82). The shift towards personalised, real-time navigation in hospitals means wayfinding systems as a resource must become amenable to automation (*ibid*). Gradually, as healthcare services have integrated wayfinding through a hybrid of digital and physical spaces, ranging from online simulations of hospitals to kiosks and digital wayfinding applications (83) continuous upskilling is also required to meet the accountability requirements of the user's positioning.

### 2.3 Searching for user-based wayfinding efficiency capabilities

In considering a user-based wayfinding approach, it is challenging to distinguish the sensory information value of the system and the process. As technology-based initiatives have fuelled research (84), a systems theory approach is subjective to uncertainties that “detach the user from the environment” (85). One must question the many studies that explore a user's positioning capabilities virtually. From Kalantari et al. (65), who use virtual reality for wayfinding pre-construction testing, found limiting behaviour responses not precisely mirror real-world experiences. Contrasting, Murray et al. (86) showcasing interactions in navigating virtual cities, enabling participants to identify real-world properties and expectations. Furthermore, Merali and Davis (87) theorised that the computational nature of information in hyperspace should be leveraged to enable people to explore knowledge and remedy difficulties found in the physical environment. Increasingly, studies verify that if users become lost in cyberspace, this behaviour is likely to be mirrored in physical space (39). All forming a critical body of correspondence and raises questions about whether automatic direction information can provide “a correct impression” for users. While research is being done in this area, the contemporary academia deliberations decrease the risk of alienating users from the cognitive activities that promote brain engagement and catalyse movement. Traditional wayfinding processes are often characterised by a maximal number of search responses and spatial parameter functions between the user and the information obtained (88). Highlighting in complex settings such as healthcare setting to raise fluctuating temporal restraints in user wayfinding capabilities.

Furthermore, Brunye et al. (30), Dalton et al. (89), and Guidice (90) deem wayfinding behaviour as analogous to cognitive processes related to spatial navigation. While different methods of obtaining environmental information is via sensory filters and has become relevant to the neurological processing used to generate diverse experiences. Devlin (12) argues, “wayfinding systems modify human behaviour via visual, audible, tactile, and cultural cues within a user's experience”; thus, fluid transitions from one destination to the next are enhanced. Measuring the overarching premise of behaviours that cannot be meaningfully mapped and assessed without situating them within the experiences that lend them significance. For example, the multifaceted development of a user-centric approach in hospital design allows for a comprehensive understanding of human behaviour within built environment. Yet, prioritising user experience does not preclude efficient behaviour; rather, it enables more nuanced interpretation without reducing users to mere decision-making automatons (91). Integrating adaptive systems and differentiating design strategies proposes diverse user needs, alongside the evolving environmental constraints are accounted for – and enhances a virtual reality testing space. With insights into user movement, patterns through the hospital's wayfinding system and improvements into user experience inform customised care can be made. Alongside further investments in such outcomes are especially crucial today, people increasingly spend their lives indoors in comparison and increasingly depend on our healthcare systems (17). Ultimately, infusing the concept for sensory information within built environments as vital for fostering acquired well-being (92). Forming a symbiosis in wayfinding as a discourse between physical and digital spaces, leveraging mix capabilities to create traversal environments (93).

Consequently, in the realm of therapeutic user-based wayfinding, individuals rely on multiple sensory processes to assess their surroundings, make decisions, and respond. Fundamental as a survival mechanism and generally aligns with growing cognitive neuroscience literature that underscores the multisensory nature of experience, specifically (94–97). At the forefront of social, cognitive, and emotional development within built environments, the integration of sensory information and responsive mechanisms is particularly crucial in unfamiliar spaces. To manage the stress of unfamiliarity, individuals employ a range of adaptive and defensive behaviours, often referred to as a fear-mediating mechanism (98). Stemming from the innate human drive for self-preservation, as a field it is rooted in the ecological aspects of wayfinding (99). Therefore, biologically adaptive systems have the capability to express coping strategies, such as the well-known fight-or-flight response, which can also manifest as a freezing strategy (100). As well as, responses are modulated by cognitive understanding, influenced by an individual's biological determinants of resistance to stress-induced diseases (*ibid*). In this context, there is a need to innovate and employ therapeutic computation within wayfinding systems to build coping mechanisms inspired by user biology. Including the diverse changes found within wayfinding behaviour and yet within complex environmental constraints by affording technology-enabled wayfinding to determine location and desired destination. However, moving forward research providing additional context is suggested on the user to aid this process, with additional enhancing virtual reality testing growing in relevance. The opportunities in spatial technologies can evolve from static information management systems to user-aware systems and advance personalisation for different abilities for different spaces and at different times.

## 3 Justification of research and method

Crafting the text, a paradigm shifts towards a user-centric wayfinding model in healthcare facilities presents as a starting point for continued user-based healthcare wayfinding research. As a narrative review, limited stipulation here has been given to a standardised structure (101). However, to aid in the study selection, the need for a narrative review identifies useful to widening accessibility literature selection while “reducing the risk of a suboptimal reporting” (*ibid*). To optimise the user's wayfinding potential in a neurophysiological communication dimension, the above literature search contribution displays a dynamic process from ecology to aid our understanding of healthier wayfinding user movements. Forming across three key areas; from physiological such as anxiety (3, 5, 7–9, 102), space confusion (14) and disorientation (68, 69) to cognitive including processing (6, 10), bias (103) and coping (99, 104–107) to explore ways to operationalise equity in behavioural factors significantly for exploring information availability (34, 55, 56, 62, 67, 76, 108), environmental hazards (15, 104) and physical mobility (109).

The practical pressure of this literature's need is reflected from the NHS (110) released call to reduce the thousands of missed appointments each month. From 2021/2022, 7.8 million appointments were missed and an average of around 650,000 a month. Contemporary wayfinding tools contribute to healthcare appointment scheduling inefficiency as costs spent in hospitals from late appointments and no-shows have increasingly affected the healthcare bottom line since the 1990s and now impact the waiting list. Where the NHS face the longest waiters in time, reaching 18 months as of November 2022 (14–16, 110), it requires exploring the limitations and legalities of user awareness levels in relation to tailoring dynamics in part of the reasons as a potential wayfinding concern. In 2011, it was documented that over a period of 8 years, 25 UK hospitals developed wayfinding strategies based on the existing NHS Estate guidance; however, staff, consultants, along with observational work indicated all sites had major wayfinding problems (93). Each site documented varied causes, and therefore, the solutions differ without autonomising through digital interventions, critically implicating, spatial-temporal solutions have yet to be implemented. By synthesising ecological reflections to personalising experiences as an alternative wayfinding approach, the relevance of contextual behavioural segmentation demonstrates as worthwhile research for root causes behind non-attendance (111). Ultimately, we argue that any investments made to healthcare wayfinding hereafter should reflect a nuanced understanding of the diverse needs and neurological factors affecting healthcare users.

### 3.1 Theories used for developing an ecological wayfinding

Adapting on wayfinding ecology implications, three following inter-connected models form the conceptual framework for a therapeutic user-based wayfinding system to best present current available evidence for wayfinding positioning for all to access healthcare settings.

The first framework is based on the space time continuum (112) and is commonly used as an approach fusing space alongside the dimension of time and is critical to possess the potential to manipulate the built environment experience. It is deemed an appropriate framework for time studies in the built environment and for urban planning professionals as a tool to develop the ephemeral nature of motion, to house memories and sensory experiences (113–118). A healthier user-based wayfinding development identifies the elements that require such a causal relationship across the two dimensions. From the outcomes of uncovering cognitive distinctiveness in users for healthier wayfinding are to be based on the understanding that the lower the awareness (legibility of the space), the higher the temporal information required. Part of human experience, time is not achieved by an isolated and momentary motion but to the continuum of movement. Highlighting value to transform wayfinding reviews and mitigates the emotional or perspective bias through physiological responses. Critically helps a user internalise space in line with the pace of memory recollection. Simultaneously, most of the wayfinding literature examines selected causal relationships (104) sourcing sensory spatial information to aid a user's real-time neurophysiological needs, dependent on measuring how users react and respond to acquire spatial legibility. Ultimately denoting environmental space to not be static, information can be reflected at different times, which is required for different people.

Secondly, without assuming everyone's cognition is the same, the framework Human Activity Assistive Technology (HAAT), is from an occupational health and ergonomic perspective to understand hospital users' wayfinding at a spatial to temporal level. The process used builds on a nuanced understanding of the diverse needs and neurological factors affecting healthcare users. Adapting the novel model with HAAT, establishes reasoning in optimal interaction, based upon guiding user-based wayfinding assessments and aiding a prescriptive forecast for successful interpretation of information outcomes. Additionally, proposed as a method to be used beyond the assistive technology phenomenon, the World Health Organisation (WHO) discuss all people are to be assumed to have an impairment at one point in their life (119). The HAAT model in 2002, is an identified emergent that WHO drew relevance to in the context of aiding new definitions for disability and handicap (120). Both connoting that the HAAT model should be diffused in articles beyond the search terms “human activity assistive technology” and integral to the design of wayfinding services not an add-on offering for selected users.

Across a spatial-temporal overview of the user in the first framework with then accessing the model in the HAAT, the activity component within HAAT is “discerned from the personal meaning to exploring why and how the activity is performed” to be motioned (121). Rather, than connecting to the space alone, the physical, cognitive, emotional, and attitudinal abilities become synced to the activity (122). Its application for sourcing a healthier wayfinding system becomes rectified to identify user types, which outline their activities in the context of healthcare infrastructure. As well as aids healthcare infrastructure to consider accessibility *for all*, including the psychology behind the human dimension of people. Looking on one hand at the anatomical user of healthcare, a change in mapping navigation is step by step and could be planned from top-down requirements. Including user type (for example, patient or staff) and transitionary needs (for example, age determents from adult to elderly user group. according to the NHS). To the other hand, with the physiological user needs of healthcare infrastructure, gaining a commitment to people management typically comes from a bottom-up approach (123). Therefore, information cues can summaries improvements that consider the sensory, safety and ability to changes.

Lastly, the curb-cut effect reiterates the importance of wayfinding in healthcare based on a set of multi-model activities to establish the ways of thinking for sustainable equity in wayfinding systems. The curb-cut effect typically has been referenced from how the evolution of side ramps that graded down sidewalks connect adjoining streets benefited from a wider range of users than solely wheelchair users. Such as strollers, cyclists, delivery workers, luggage holders, running/ walking, and skateboarders advocating a tools usage for a wider population in diverse activities. Mindful to wayfinding, Prandi's (124) systematic review for indoor wayfinding and navigation accessibility found 44 articles on assistive support for wheelchair users (ramps added), blind users (audio instructions supported) and those new in an environment (beacons at decision-making points including doorways, corners, room interiors, intersections of hallways. However, Chang and Wang (125) highlight, whether these accessible solutions support others is not shared. Currently, wayfinding methodologies aid in navigating across healthcare infrastructure including factors from user-types that counteract principles for effective wayfinding. For example, a new staff member may be asked for directions from a patient, or a visitor may take an elderly patient through a long corridor without seating. Alas, curb-cut effect embeds the forward-thinking view to wayfinding and propositions for the wayfinding model in its development.

Ultimately, by separating wayfinding users and their activities from both spatial and temporal measures, a greater number of users can use healthier information cues according to their needs. Synthesising the curb-cut effect with the space time continuum and HAAT ensures verifying ways disability-friendly features are accessed and utilised by a wider group than the people it was designed for. However, the collection performance of various activities across user diversification in the healthcare context is limited and restricts sustainable reporting frameworks and practices in wayfinding studies moving forward – which is the focus of this framework for future research developments in therapeutic user-based wayfinding for healthcare.

## 4 A therapeutic user-based wayfinding system in healthcare infrastructure

The identification and understanding of a therapeutic user-based wayfinding system is crucial for enhancing user experience and satisfaction in healthcare facilities. In this section, we explore various factors that play a role in determining the needs and preferences of users in wayfinding. Particular attention must be given to the heterogeneity of users, their experience preferences, and their specific characteristics and requirements to develop an effective wayfinding system. Furthermore, consideration of alternative informational needs, such as multisensory experiences, embodied experiences, disability-friendly design, and fluctuating capabilities, is imperative to ensure inclusivity and accessibility for all users. By considering these confounding factors, we can create a user-based wayfinding model that addresses the diverse needs of healthcare users. Thus, this initial framework serves as a blueprint for identifying behavioural led, wayfinding systems to be examined through the lens of the nuanced factors that shape healthcare experiences.

### 4.1 Conceptual framework: integrated user-based wayfinding model

**Figure 1:**
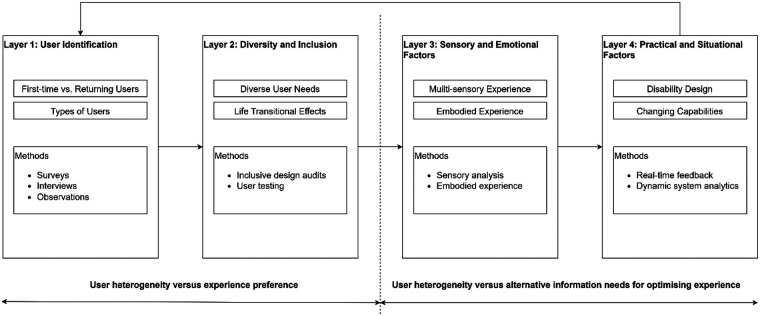
Integrated user-based wayfinding model.

Figure 1 illustrates various factors influencing the wayfinding experience in healthcare facilities. It highlights the importance of considering different types of users, including first-time and returning users, as well as inpatients, outpatients, visitors, healthcare staff, and service providers. The figure emphasises the diverse needs of users, such as those with sensory loss, neurodiversity, physical impairments, sociocultural differences, and caring responsibilities. It also showcases the temporal impact of life transitional events on wayfinding, including age-related changes, COVID-19, stress, chronic pain, and mental health. Expanding into its synergy for the user-based wayfinding, the figure further emphasises an information need to be diverse as well. Highlighting the significance of creating a multisensory experience and considering embodied aspects of wayfinding, which include emotions, sociocultural factors, and previous experiences. By consolidating the importance of disability design alongside accessible wayfinding in compliance with standards across the wayfinding journey, thereby meeting the needs of differently abled individuals. Lastly, stressing the importance of informing rather than directing users implicates the need to address frustrations and concerns related to wayfinding that ensures a person-centred approach in healthcare facilities. In totality, this model could serve as a means of testing performances by end users to verify or accept a wayfinding system as user-based, in alignment with the UK NHS healthcare objective for a patient-centric approach.

### 4.2 Identifying and understanding a therapeutic value in user-based wayfinding system

In healthcare facilities, effective wayfinding is integral to ensuring a positive user experience. Nevertheless, the diversity of users' needs and characteristics introduces complexities in designing inclusive and accessible wayfinding systems. This section aims to explore user-centric approaches in healthcare wayfinding, taking into consideration various user factors and requirements.

(I) First-time vs. Returning Users: A well-established practice for wayfinding exists aimed at aiding first-time healthcare users. It is commonly assumed that if an individual can successfully navigate during their first visit, subsequent visits should present no issues (45). Contemporary healthcare design considers first-time users, who often possess little to no prior knowledge compared to those with preconditioned familiarity. Conversely, returning users necessitate up-to-date information to orient and reorient themselves amid any changes such as renovations, new additions, or reconfigurations, could affect their ability to navigate without confusion and spike disorientation beyond a preexisting cognitive map. Given these contrasting needs, gathering information about if a user is a new or returning user would facilitate integrating safety, accessibility to personalising their visit based on awareness needs.
(II) Types of Users: Discerning whether a user is an inpatient, outpatient, visitor, healthcare staff, or service provider would enable the identification of distinct needs to be met within the healthcare facility, both spatially and temporally. This information is also beneficial for navigational purposes among future users (126). Various patient groups have diverse needs (127), and eliciting patient contributions to wayfinding can enhance healthcare services (126, 128). While, inpatients require specific room directions, treatment information, and amenities, outpatients differ and need guidance from clinics, departments to support their appointments. These needs often come with the added complexity of stress or anxiety moving between clinics and contrasts the requirements of visitors, who mainly need information about waiting areas and parking. As healthcare staff must navigate quickly to minimise delays and disruptions, while service providers need a thorough understanding of the facility and any subsequent changes.
(III) Diverse User Needs: Users with various needs such as sensory loss, neurodiversity, physical impairment (129), sociocultural backgrounds (non-english speakers) (130, 131), and caring responsibilities may require different types of information as well as delivery. Reducing feelings of isolation and fostering a sense of belonging Souza and Martin's (129) systematic review found that feelings of respectfulness, inclusiveness, and acceptance by the environment are crucial for these user-centrism. The variations between such users may encompass differences in information processing, overstimulation levels, and preferences for alternative communication modes such as picture-based or multilingual communication.
(IV) Life Transitional Effects: Life events such as reaching a significant age, the impacts of COVID-19, increased stress levels, long-term chronic pain, and changes in mental health can lead to a period of instability. The concept of “unanticipated transitions”, which relates to underdeveloped cognitive coping mechanisms, has been described as having both psychological and physiological consequences that affect the user's ability to effectively navigating a building and adapting to daily life changes (104, 106). In the context of wayfinding, cognitive bias can be found in longer walking abilities; to access to additional travel support which would change in the ways for improving the route developments in the systems. Including the role for wayfinding to aid and impact mnemonics form as does the need to adapt wayfinding systems to better accommodate these transitions.
(V) Multisensory Experience: Although wayfinding has traditionally been considered a primarily visual activity (132), the utility of incorporating alternative sensory information is becoming increasingly recognised. With clear acknowledgement, multisensory and digital communication can aid in delivering a continuous analogy through audio, haptic, and cross-sensory experiences. Arthur and Passini (133) contends that the human body can trigger sensory information through various channels, such as sound and touch, thereby offering users more direct, unmediated access to information.
(VI) Embodied Experience: Since, conventional wayfinding focuses on finding the shortest route between two points (29, 134), the embodied experience in healthcare considers previous experiences, emotions, and feelings. Specifically guiding users in orienting themselves towards (and away from) safe spaces, blind spots, and environmental artefacts (1). Decisions about routes often encompass considerations of comfort and safety, reflecting the user's “corporeal and cognitive states” (1). However, the experimental nature of wayfinding today, has yet to be fully explored in this context.
(VII) Disability Design: Design for accessibility, or “accessible wayfinding design”, considers the distinct informational needs required for diverse abilities. This approach not only overlaps with sensory information but also extends to include features such as size, shape, and contrasting sets of cues, including audio volume and space accessibility. deriving from the Web Content Accessibility Guidelines (WCAG) international standard stipulate that technology, and infrastructure must comply with WCAG 2.1 standards and cover many of these elements, within digital wayfinding access as a legislative requirement (135, 136).
(VIII) Changing Capabilities: Ultimately healthcare facilities serve as spaces for interaction, necessitating a focus on informative rather than directive wayfinding cues. This user-centric approach values the individual's experience over mere efficiency requirements aiding in wayfinding value to the environments influence on mental and emotional wellbeing. Furthermore, users despite encountering difficulties in navigation, have referred to feeling too embarrassed to ask for help or may not wish to inconvenience others (12, 43). Following the means of perceived stress on wayfinding outcomes implicates the holistic overview to physiological implications in wayfinding correlated to factors such as frustration and embarrassment and in turn affecting the healthcare facility's ability to provide person-centred care.

### 4.3 Synthesising user-based wayfinding into the UK healthcare settings

Exploring wayfinding behaviour from neurophysiology and benchmarking it against wayfinding healthcare standards 1990 and 2005 demonstrates a theoretical and practical gap in understanding changes in wayfinding, despite advancements across a digital landscape. Inferring what landmarks mean, why they are used and how signage adds value to the wayfinding experience are now critical to a diversified systems benefit for wayfinding's adoption.

**Figure 2:**
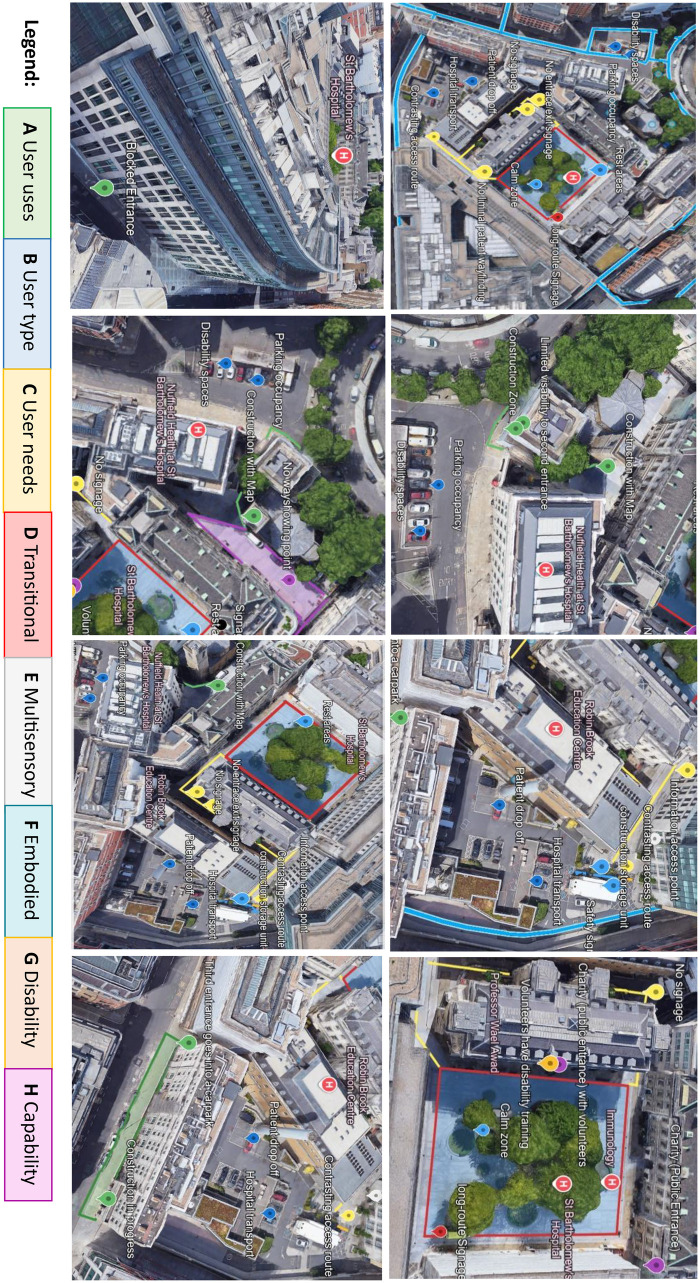
Spatial scanning activities mapped using the user-based wayfinding model across the case-study site. The figure presents aerial and oblique views illustrating key wayfinding dimensions: A) User cues, B) User type, C) User needs, D) Transitional spaces, E) Multisensory factors, F) Embodied experience, G) Disability, and H) Capability. The legend is embedded within the figure, with colour-coded overlays integrated into each image to denote the corresponding dimension. This mapping highlights the relationship between user diversity, experiential factors, and spatial configuration within the built environment.

The user-based methodology required is to refer to the removal and exclusion of individuals that avoid adverse events in the case of potential movement disablers. The user-based wayfinding model paves the way for the adoption of technological advancements in the study of wayfinding, within the realm of the healthcare-built environment. While Figure 2, shares the novel reference thread by using the eight categories and comprises the framework under two dimensions from a spatial to temporal perspective and how might personalisation advance the healthcare environment. We therefore argue that wayfinding healthcare standards also require updating to aid in the growing personalisation of wayfinding research Following a view to long-term patient-centric priorities and the consequences in which healthcare resources are used, analysed from an ecological perspective to deem a therapeutic, user-based wayfinding system to be long due.

Below is an example of St Bartholomew's, a UK healthcare PFI building, with high level visuals taken from Google Earth and wayfinding mapping information to understand the complexities of the infrastructure. Its current state is undertaking major construction work and therefore a lot of entrances are blocked without adequate signage. However, as a cancer specialist centre, many outpatients travel from out of the hospitals vicinity to receive treatment. Based on this context, there is potential for an array of different activity-led interpretations from the framework to consider the different users to be accounted from systems of wayfinding information and yet to be afforded with. The proposed approach identified in the figure's legend considerations, leverages a holistic demonstration in how future technological advancements can shift wayfinding understanding and action.

Using a combination of frameworks including 1) the integration of the space-time continuum, to support how space is used in part of environmental and user changes. 2) The implementation of the HAAT model based on human and activity features into a therapeutic healthcare wayfinding technology that prioritises a synergy between what activities in a healthcare environment. Considered based off the diverse needs of a population and responsibilities of a user and 3) applying the curb-cut effect methods with accessibility in the design process. The following analysis in this section considers how to innovate the lack of wayfinding user heterogeneity in contemporary wayfinding and source alternative ways for information advancements that can be modified to support a therapeutic user experience.

**Figure 3:**
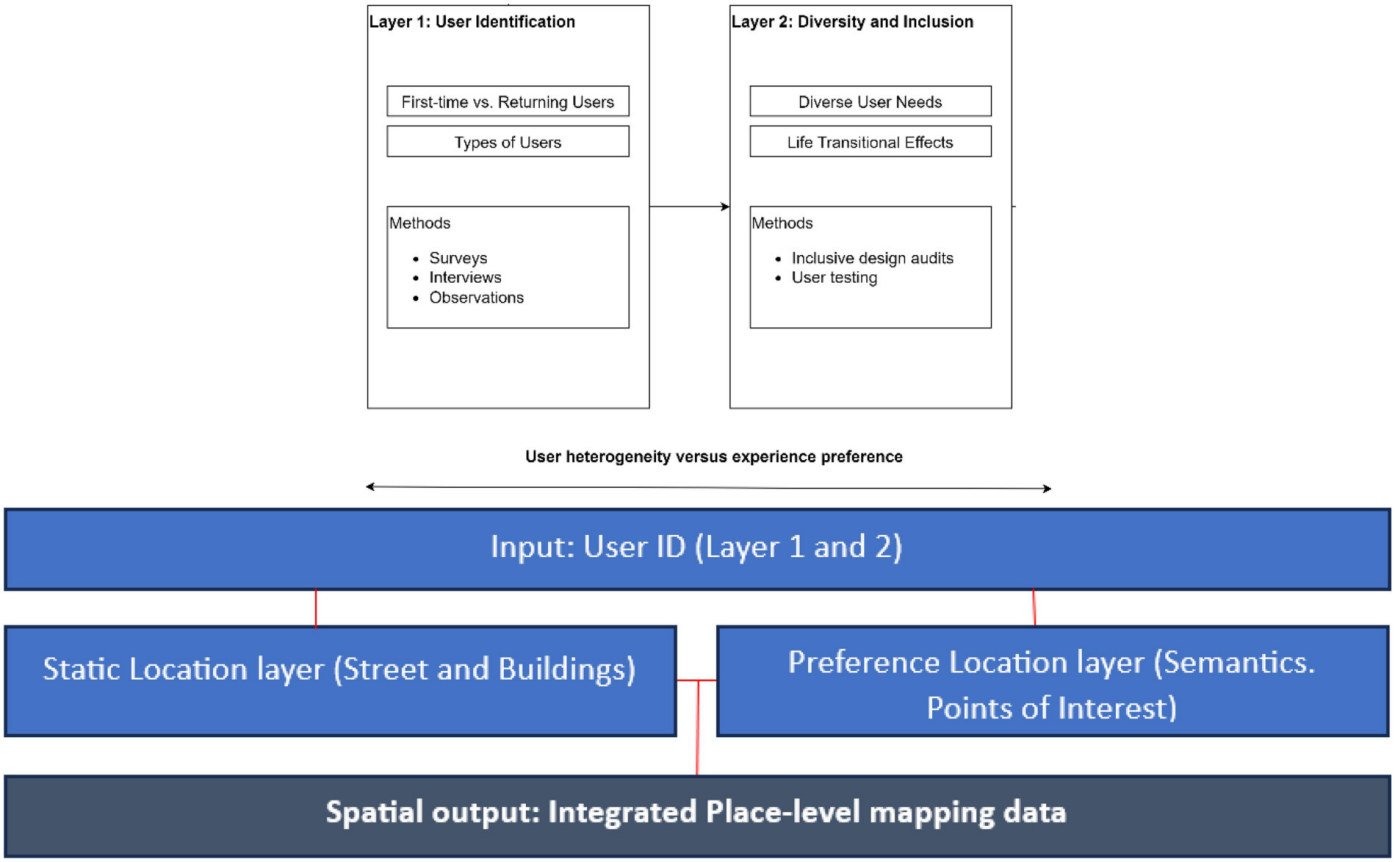
Integrated human place data codes for user-based wayfinding model.

**Figure 4:**
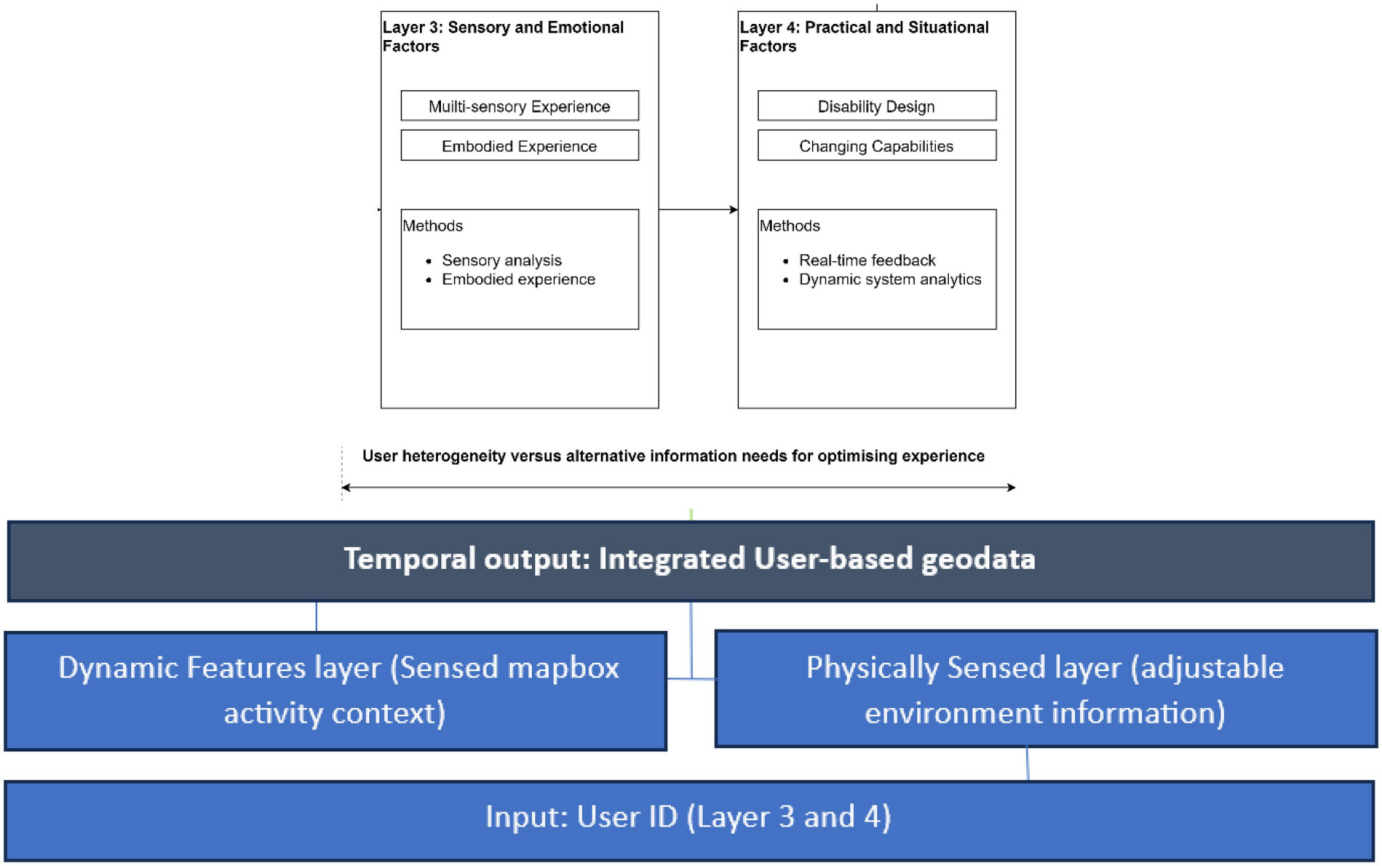
Integrated context-specific environmental geodata for user-based wayfinding model.

Ultimately, ensuring that such digital wayfinding information is managed, supports a group of contrasting users based on how their activities physiologically connect is hypothesised. Specifically, how layer 1 and 2 (categories A-D) interact with space and layer 3 and 4 (categories E-H) interact with time in order to support accessibility to all healthcare users, reflected in Figures 3 and 4. However, some limitations are indeed reviewed, concluding with a roadmap for future research and delineating how each task aligns with pertinent supporting materials.

#### 4.3.1 User heterogeneity vs. experience preference

As a user's thought process plays an integral part in their orientation to navigate but also to orient in the healthier environment, enforces how efficiently users can engage in wayfinding through a static map. Taken from 2024, St Bartholomews main entrances are blocked, covered by scaffolding and creating blind spots around the outer area. The constrain being that if you are an existing user, then subconscious mapping would have been considered, whereas to a new user the entrance would not necessarily be clear. Furthermore, visitor parking is in a different space to patient drop off which is aligned with the staff facilities entrance. Both parking facilities positioned within side entrances require wayfinding information for using the closer entrances over the main entrance and reducing walking to destinations. Furthering limited or longer route signage ultimately impacts different user needs, particularly in the square at each point creates added strain of finding destinations. Without considering landmarking support, which direct users from point to point and results to an indirect routing required.

In contrast to the direct route mapping experience from the cognitive processing perspective (137–140); these studies have complimented studies for user abilities to demonstrate tangible differences (140, 141). What's found is that for a successful wayfinding experience across a diversity of users, environmentally centred cues (specifically a piece of information regarding the landmark) must be positioned and represented in a way that is legible to the user as a spatial feature. However, how would we know if the cue is legible during wayfinding orientation? By breaking down the form of a user to identify spatial and temporal impacts of the space, between categories A-D optimises solutions that could be provided for overall user satisfaction, as well as understanding how these factors determine the needs of the user and interplay for thoughtful decisions that enhance the user experience.

With this level of understanding derived from user-environmental semantics, using technological computation, I) the code of the landmark, formed into an abstract indicator within a map as a “place”, can be used to orientate and then used together to create distance. Evidence in Yesiltepe et al. (142) review has indicated that part of the information regarding the prominence and proximity of these cues, if correctly encoded, can form cognitive mapping. Consequently, Epstein et al. (143) stated that cognitive maps complement map-like spatial codes when they preserve “entities that are closer together (vs. farther apart) in the real world are close together (vs. farther apart) on a map”. Therefore, a spatial technological system would be able to first leverage landmark cues with this user data and then, predict the type of temporal information required based on the pressures that the varied users face. As diverse healthcare users, their experience should be mapped by cues and understood with the distance that is legible between each code of the landmarking.

#### 4.3.2 User heterogeneity vs. alternative information to optimise the experience

While users can navigate without landmarks and any path integration, a method known as landmark-based piloting, their inclusion into wayfinding information tools has strong potential as controllers to correct user spatial errors (144). Landmark anchoring and environmental boundaries act as basic cues for orientation in a map when users are lost. In these circumstances, cues are there to help users react and recover their sense of direction by “connecting map coordinates to fixed aspects of the environment that can be identified by perceptual systems” (143). When looking into Figure 2, there are opportunities to enhance some of the sensory capabilities of the hospital such as, informing users where and what to seek at the information access point, at what location disability trained volunteers reside and places to seek solitude and rest. There are also blind spots between buildings without any signage and amid confusion difficulty in user navigation capabilities to arise. Being cautious to spaces as “zoned areas” of a hospital and how they emerge at the diversified experience level would be an approach to understanding how best to recover the wayfinding services and unravel intangible information from categories E-H.

In this case, by digitally mapping the positioning of landmarks supports a response to objects that are in less navigational decision points, thus being positive for aligning a cognitive map and for determining the stability of potential landmarks. At the crux of developing and forming the different information needs for wayfinding behaviour and how their needs change over context would also be reflected. Therefore, to establish the directional route mapping for the cues (specifically depending on the type of route such as safe, aesthetic, or open space route), user wayfinding experience should accommodate such preferences within their landmark anchoring. In this case's example, an outpatient undertaking cancer treatment who realises an alternative route where they can rest on a bench mid-way through a hallway demonstrates both preconditioned and reactive information is relevant. In times of recovery from the sense of direction, different information types could be preferred for specific user needs.

Furthermore, by continuing to understand the information mode of requirement needs during user shifts in orientating space and places better emphasis on the user's control for II) planning a route to a user's destination and identifying useful shortcuts is proposed. While there is usually more than one route to a destination, recent studies explore how virtual outdoor wayfinding shows a topological connection of street views that provide accurate spatial data surrounding the user's location for re-routing and pre-planning (142). Recognising that there are increased route options that provide a wider choice of global connectivity to the rest of the mapping network, also identifies the opportunity for off-route landmarking of decision points, also known as in-leg points of interest or areas of interest that can provide cognitive-aiding and confirmation to users that they are on the right route (*ibid*). Inspired by outdoor urban planning rather than a turn-by-turn route, encapsulates wayfinding cues from the person of interest's information needs and the type of information available on the way becomes context-environment specific, contrasting to the environment remaining siloed from user behaviour. Ultimately, this research requires further expanded knowledge in the built environment, specifically regarding complex healthcare building adaptability and findings.

### 4.4 Limitations

The integrated user-based wayfinding model was developed to be generic to all forms of technologically innovative wayfinding deliveries. While this is to afford a foundation for assigning user-based confounding factors, determining variegated needs between the user, the information and the healthcare facility raises some complexities that interfere with formulating the understanding between the components and forecasting. As an initial response to strategic wayfinding capabilities, beyond this review, further information is required to address if a personalised wayfinding system is required in replacement or to complement a universal wayfinding system. Specifically, basing it on the subjective judgment about the patient and wider user abilities to independently reach their destination.

Here, we suggest, lack, and question whether access can or cannot be guided by a set of universal standards that devalues the lives of individuals. However, since the NHS hospitals use private sources of money to pay for the upfront costs of their design, build and maintenance, changing the physical space may be constrained. While adding a digital interface may also add to information overload as well as structural wayfinding system errors, it all needs to be considered prior to installation. For example, Bubric et al. (145) showed that the position of cameras, curtains, and bulkheads is not considered within department floor plans and therefore results in additional signage changes to the space following the evaluation prior to installation. Also, with dynamic wayfinding considered for healthcare wayfinding solutions to show real-time information, a connection between the construction strategy and aiding users to navigate a changing environment is required.

Other services would also need to be considered within the operations process. From queries to adopting personalised wayfinding, the extent of other wayfinding tools leading to ill fate due to the diversified signage directly correlates to reviewing safety and efficiency regulations. From the user's perspective, temporal levels of information management follow into further exploration for strategies on planned and unplanned visits for users who require healthcare assistance tailoring wayfinding solutions regardless of their level of preparation should be further explored. Wayfinding indeed crosses over to the maintenance of health and safety, emergencies, and instructions within a healthcare setting. Further research is required beyond this study to explore emergencies at a neurophysiological level, particularly with changing UK construction safety regulations, like the UK building safety act (2022).

While joined-up thinking across systems is required, controversies surrounding the operationalisation of models in digital healthcare systems are deeply intertwined with their implementation to convert indoor universal systems and the economic investment value. Short et al. (149) explored patient-centric solutions across different departments of the hospital; however, they found local small-scale problems led to a small organisation-wide impact. However, wayfinding design features are not an integral part of the architectural design process (12). At that point of considering healthcare surveys and observation techniques for systemic level changes, it is argued “to come from approaches proven elsewhere, due to high financial investment and associated risk” (*ibid*).

The different needs associated with user-based wayfinding resides in the interventions for primary research and secure protected characteristics. Predominantly, understanding how digital wayfinding techniques can inform implementation would yield in the model's verification. Unrequited disclosure remains data deficient and remains subject to ongoing privacy issues, such as the sharing of data with the aggregator, leading to the disclosure of privacy as examples. Thimbleby (146) argues, that no predictions can satisfy everyone, although it is salutary that many divulge personal information as people want to lead healthier lifestyles. From a humanistic perspective, it is extremely important that research further explores the quantitative, engineering, and computational sensibilities that prevail beyond the limitations of this review, which is subjected to systematic critique and explores the trade-offs taken by different data-sharing methods.

Overall, the integration of a user-based wayfinding model requires infrastructure coordination among diverse stakeholders, which can be resource-intensive. The actual deployment of such systems at scale demands meticulous planning, computational resources and data privacy considerations requiring more research to be done in the area. For NHS England to enhance, the quality and accessibility of healthcare data should be underscored by collaborative efforts with the Department of Health and Social Care. A therapeutic user based wayfinding model is a step in the right direction for exploring wayfinding tools for user healthcare infrastructure equity. However, the issues relating to the model's scalability, interoperability and resource allocation are acknowledged, and the macro-level challenges require ongoing research, collaboration and iterative development to refine and optimise the model for real-world application.

## 5 Conclusion

The prevailing challenge in healthcare facilities centres on the creation of a wayfinding system that is not only efficient but also empathetic to a diversity of user needs. Conventional systems often lack the nuanced understanding of various user categories, including first-time and returning users, inpatients, outpatients, visitors, and healthcare professionals, as well as those with specific needs such as sensory impairments, neurodiversity, and physical limitations. Moreover, these systems rarely account for life transitions or sociocultural differences that influence wayfinding behaviour. To address this complex web of factors, this research introduces a pioneering conceptual framework for an integrated, user-based wayfinding model. Unlike traditional models, user-based wayfinding with a therapeutic lens is attuned to the myriad needs of healthcare users. It considers not just the demographic and physical characteristics of the users but also delves into the temporal shifts in their lives, such as ageing or stress-related changes. Furthermore, it encourages the design of multisensory and embodied experiences, emphasising the inclusion of disability-specific designs and compliance with accessibility standards. By dissecting the individual and collective factors that influence wayfinding in healthcare settings, this review offers a comprehensive blueprint for a new generation of wayfinding systems. It aligns with healthcare objectives that prioritise patient-centricity, inclusivity, and accessibility, thus acknowledging future-proofing the technological enhancements in healthcare facilities, the limitations can be directed. Ultimately, this research lays the foundational groundwork for ushering in a transformative change in how we understand and implement wayfinding systems, prioritising healthcare facilities however can transcend to all complex built environments that require nuanced ecological information and management.
